# COVID-19 made it harder to access period products: The effects of a pandemic on period poverty

**DOI:** 10.3389/frph.2022.1003040

**Published:** 2022-11-10

**Authors:** Emily Hunter, Kirstin Palovick, Mintesnot T. Teni, Anne Sebert Kuhlmann

**Affiliations:** College for Public Health and Social Justice, Saint Louis University, Saint Louis, MO, United States

**Keywords:** COVID-19, period poverty, period product insecurity, pandemic response, work absenteeism, menstrual hygiene management

## Abstract

**Background:**

Prior to the COVID-19 pandemic, a few studies started to highlight the extent of period poverty in the U.S., especially among low-income women and girls. Preliminary data documenting the effects of the pandemic, subsequent economic downturn, and closure of schools and businesses on menstrual hygiene management are now emerging.

**Objective:**

This study explores the relationship between the effects of the COVID-19 pandemic and period poverty among a nationally representative sample of U.S. adults.

**Methods:**

Cross-sectional, secondary analyses of a 2021 nationwide, self-administered, online panel survey used weighted logistic regressions to assess the relationship between the COVID-19 pandemic making it more difficult to access products and missing work due to a lack of products. Responses from 1,037 menstruating individuals age 18–49 were included.

**Results:**

Overall, 30% of the sample indicated the COVID-19 pandemic made it more difficult to access period products, 29% struggled to purchase period products in the past year, and 18% missed work due to a lack of period products. Those who identified as Hispanic (aOR 2.06 95% CI 1.29–3.29) and had children under 18 (aOR 15.3 95% CI 1.03–2.26) were more likely to indicate that the pandemic made it harder to access period products. Subsequently, those who indicated that the pandemic made it more difficult to access period supplies were more likely to report missing work due to a lack of period products in the past 12 months (aOR 4.32 95% CI 4.69–6.94).

**Discussion:**

The COVID-19 pandemic exacerbated period poverty, especially among those in the U.S. who struggle with accessibility and affordability of products. Future pandemic response planning should consider period products as a basic need for vulnerable households. In addition, policies that increase the affordability and accessibility of period products for all should help reduce menstruation-related absenteeism from work.

## Introduction

For several years, public health literature has focused on menstrual hygiene needs and the impact of unmet needs on women and girls in lower-income countries (1). Now, literature is emerging from high-income countries as well, such as New Zealand (2), Spain (3), and France (4). In the U.S., women and girls also face challenges accessing needed menstrual hygiene products and the privacy to change them regularly (5, 6).

The American Medical Women's Association defines period poverty as, “the inadequate access to menstrual hygiene tools and education, including but not limited to sanitary products, washing facilities, and waste management” (7). Prior to the COVID-19 pandemic, some of the first data from the U.S. documenting the extent of unmet menstrual hygiene needs among low-income adult women were published (8). In addition, data were collected prior to and then published during the pandemic that showed the magnitude of period poverty among high school students and the extent to which they relied on resources available at school to access period products (9). At a Missouri high school, where 99% of students are eligible for free or reduced lunch, around 60% of girls indicated they obtained period products through their school and 48% reported that at least once during the past school year they needed products but did not have money to buy them (9). A 2019 survey documented period poverty and the subsequent effects on mental health among college students (10) while another 2019 study found an association between lack of access to period products at school and students' ability to learn (11), but data about the general population are still limited.

Efforts to alleviate period product insecurity have gained momentum over the past few years. An increasing number of public schools, universities, prisons, and workplaces provide free menstrual hygiene products as a result of recent policy changes (12). Progress is ongoing, however, as only 17 states and Washington D.C. currently require menstrual products be provided in public schools, and 24 states still tax them as luxury items (13). Diaper banks, food banks, prisons, and homeless shelters are increasingly distributing free menstrual hygiene products, but they often rely on donations which may limit the consistency and variation in their supply (14). Furthermore, these relief efforts are not available to all women, as most require in-person interaction to obtain assistance and access to these locations can be hindered by numerous barriers, such as inconsistent supplies at facilities or “gatekeepers” who provide small numbers of products upon request only (5).

As the COVID-19 pandemic hit the U.S., schools shut down for months, an economic downturn hit the most vulnerable households especially hard, and demand at food banks and for other essential services such as assistance with rent and utilities skyrocketed (15). A diaper bank in North Carolina saw an 800% increase in the demand for period products during the COVID-19 pandemic (16); food banks around the country faced shortages as unemployment rates rose (17). Policy changes to combat period poverty were enacted when the Coronavirus Aid, Relief, and Economic Security (CARES) Act, passed in March 2020, deemed period products as “medical necessities”, authorizing the use of funds from health saving accounts and flexible spending accounts to purchase period products (18). While this classification allows use of non-income dollars for period products, individuals first must have benefits with health spending accounts, established funds in those account, and the knowledge about this new policy in order to benefit from it. Low-income, uninsured women and families are still unable to use other government benefits to purchase period products (19).

Others have called for research into how the COVID-19 pandemic has affected period poverty in the U.S. given the economic impact of the pandemic on financially vulnerable households (20). Now, data are emerging to be able to address these questions. A recent study conducted in 2020 in the U.S. found associations between income loss from the pandemic and a greater inability to afford products among menstruators who were already enrolled in a large, longitudinal cohort study about the spread of COVID-19 nationwide (21), but other data are limited. Therefore, the purpose of this study is to explore the relationship between the effects of the COVID-19 pandemic and period poverty among a nationally representative sample of U.S. adults.

## Methods

Cross-sectional, nationally representative data were collected in April and May 2021 through a self-administered, online survey that took approximately 15 min to complete. The survey was a collaboration between YouGov®, U by Kotex®, and the Alliance for Period Supplies (APS). YouGov maintains a panel of thousands of U.S. adults. Enrolled panelists receive periodic survey invites *via* email. Once they initiate the screening process from an invite, they are directed to active surveys in the queue for which they meet inclusion criteria. Surveys remain open until quotas for a representative sample for that survey have been reached. Panelists accrue points for each survey they complete through YouGov which can then be redeemed for incentives such as gift cards. The survey was administered in compliance with industry standards for market research. Respondents were asked a variety of questions about their use of and access to period products, but the specific term “period poverty” was not defined or used in the survey. Data were made available to the authors by APS for secondary analysis. Here, we present results from secondary analyses of these de-identified survey data to determine the associations between the effects of the COVID-19 pandemic and access to period products, such as tampons, pads, and liners, in the U.S., controlling for socio-demographic characteristics.

In order to restrict the final sample for analyses to respondents who indicated they were currently menstruating, inclusion criteria for these analyses were participants who had experienced at least one period in the past 12 months and were considered to be adults of reproductive age, i.e., 18–49 years old. Out of 5,178 individuals who initiated the survey, 1,037 (20%) records met these criteria. Gender was not used as an inclusion criterion.

### Dependent variables

Three main outcome variables were identified: (1) the COVID-19 pandemic making it more difficult to access period products, (2) struggling to purchase period products in the past year, and (3) missing work in the past year due to a lack of access to period products. If the pandemic made it more difficult to access products was a categorical yes/no variable. Struggling to purchase period products in the past year was recoded from a five option Likert scale into a yes/no response with strongly agree and somewhat agree recoded as “yes” and other responses as “no.” Since data collection occurred in April and May 2021, “the past year” refers to a time-period fully encapsulated by the COVID-19 pandemic. Missing work included missing either in-person or virtual/remote work in the past year due to a lack of period products. The first two outcomes, the COVID-19 pandemic making it more difficult to access products and struggling to purchase products in the past year, were also included as predictors in analyses of the missing work outcome since missing work may be a consequence of more difficulty accessing or struggling to purchase products.

### Independent variables

Demographic variables for inclusion in regression models were selected based on existing literature and bivariate analyses. Significant predictors at *p* < 0.05 in bivariate analyses for two outcomes - the COVID-19 pandemic making it more difficult to access products and struggling to purchase products - included age, race/ethnicity, education level, family household income, partnership status, and if respondents had children under 18. Household size was not a significant predictor in bivariate analyses for the pandemic difficulty and struggling to purchase outcomes, so it was not included in regression models for these outcomes. Missing work due to a lack of products had similar significant demographic predictor variables in bivariate analyses except family income was significant instead of household size so family income was included in the regression model with the other demographic predictors.

Additional covariates for all three outcomes included ever experienced period poverty personally, ever struggled to purchase products, and found period products to be unaffordable. Respondents were considered to have experienced period poverty if they responded yes to any of the following situations: (1) worn a period product longer than recommended in order to “stretch” its use because they did not have access to more period products; (2) struggled with the decision on whether to buy other basic necessities (e.g., food, soap, etc.) or period products; (3) used a substitute to a period product (e.g., toilet paper, paper towels, socks, etc.); (4) went without using period products because they could not afford to purchase any; or (5) asked someone for a period product (tampon, pad, liner, etc.) because they could not afford to purchase any. In our analyses, ever experienced period poverty refers to issues of period product access over the course of one's lifetime due to financial barriers; it does not include a lack of privacy or knowledge about menstrual hygiene. Respondents reporting ever experiencing period poverty could have experienced this before the COVID-19 pandemic, since the onset of the pandemic, or both.

### Statistical analyses

Data cleaning and analyses were completed using IBM SPSS Statistics Version 27 (22). Measures of association between demographic variables and categorical survey questions were completed *via* Chi Square Tests of Independence. A complete case analysis was performed to account for any missing data; only complete cases were then included in the regression analyses. A check for multicollinearity between variables in the regression models confirmed that the variance influence factor was less than 2, and no variables needed to be removed. Sampling weights were utilized to approximate a nationally representative sample. Multivariate weighted logistic regressions were performed using a *P* value of <0.05 as the level of significance to determine the adjusted odds ratio (aOR). As these were secondary analyses of de-identified data, we received a non-human subjects research determination from the IRB at Saint Louis University.

## Results

The majority of respondents who met inclusion criteria (56.4%) identified as white, 35.4% had some college level of education, and 39.5% were between the ages of 25 and 34. Among the final sample, 61.5% of respondents had ever personally experienced a period poverty situation, 38.1% found period products to be unaffordable based on their income, and 43.1% had ever struggled to purchase products (Table 1). Specific frequency breakdowns of each period poverty situation are included in Table 2.

**Table 1:** Characteristics of survey participants. Unweighted (*n* = 1037).

Variable	*n* (weighted %)
**Age**	
18–24	197 (18.4)
25–34	414 (39.5)
35–44	338 (32.8)
45–49	88 (9.2)
**Race/Ethnicity**	
White	588 (56.4)
Black	162 (13.7)
Hispanic	203 (21.9)
Other	84 (8)
**Education Level**	
High school diploma or less	333 (32.2)
Some college or 2-year degree	376 (35.4)
4-year degree or higher	328 (32.4)
**Family Household Income *n* = 929**	
Under $30 k	260 (27.6)
Over $30 k	669 (72.4)
**Partnership Status *n* = 1022**	
Currently in partnership	571 (57.2)
Not currently in partnership	451 (42.8)
**Children Under 18**	
Yes	441 (40.3)
No	626 (59.7)
**Household Size (*n* = 1002)**	
1–3	636 (63.2)
4+	366 (36.8)
**Based on my income, I find period products to be unaffordable**	
Yes	394 (38.1)
No	643 (61.9)
**I have struggled to purchase period products due to a lack of income/funds at some point in my life**	
Yes	445 (43.1)
No	592 (56.9)
**I have struggled to purchase period products due to a lack of income/funds at some point in the past year**	
Yes	304 (29.2)
No	733 (70.8)
**I have personally experienced a period poverty situation**	
Yes	641 (61.5)
No	396 (38.5)
**The Covid-19 Pandemic made it more difficult for me to access period products**	
Yes	291 (30.3)
No	746 (69.7)
**I have had to miss work (virtual or in person) due to lack of access to products**	
Yes	191 (18.4)
No	846 (81.6)

**Table 2:** Period poverty situations. Unweighted (*n* = 1037).

Variable	*n* (weighted %)
**Had to wear a product longer than recommended**	
Yes	351 (33.5)
**Struggled with the decisions to buy other necessities or period products**	
Yes	183 (17.5)
**Used a substitute to period product**	
Yes	379 (36.1)
**Went without products because I could not afford them**	
Yes	141 (13.5)
**Asked someone for a period product because I could not afford them**	
Yes	239 (22.9)

### COVID-19 pandemic effect on period poverty

Of the 1,037 included respondents, 291 indicated that the COVID-19 pandemic made it more difficult for them to access period products. Of those 291, struggling to afford products was the most frequently cited reason as to why the pandemic made it more difficult to access products (18.5%), more than struggling to find them (13.3%) or transportation barriers to go buy products (4%). Weighted regression analyses included 870 complete cases. A greater proportion of those who indicated that the COVID-19 pandemic made it more difficult for them to access period products are Hispanic (aOR 2.06 95% CI 1.29–3.29) or have children under the age of 18 (aOR 1.53 95% CI 1.03–2.26). Participants who said they have struggled to afford products at some point in their life were 2 times more likely to indicate the COVID-19 pandemic created additional challenges for them accessing period products (aOR 2.09 95% CI 1.37–3.19). Moreover, ever personally experiencing one of the five period poverty situations nearly quadrupled the odds of the COVID-19 pandemic making it more difficult to access products (aOR 3.96 95% CI 2.38–6.59) (Table 3).

**Table 3:** Weighted logistic regression for predictors of the COVID-19 pandemic having made it more difficult to access period products (*n* = 870).

Variable	Adj. Odds Ratio	Confidence Interval [95%]
**Age**		
18–24	1.98	0.82–4.78
25–34	2.03	0.91–4.50
35–44	1.19	0.52–2.72
45–49 (Reference)		
**Race/Ethnicity**		
Black	0.93	0.51–1.70
Hispanic	2.06	1.29–3.29*
Other	1.13	0.54–2.37
White (Reference)		
**Education Level**		
Less than high school or High School	1.26	0.75–2.12
Some college or 2-year degree	1.19	0.73–1.92
4-year degree or higher (Reference)		
**Family Household Income**		
Under $30 k	0.73	0.47–1.15
Over $30 k (Reference)		
Partnership Status		
Currently in partnership	1.39	0.91–2.13
Not currently in partnership (reference)		
**Children Under 18**		
Yes	1.53	1.03–2.26*
No (Reference)		
**Based on my income, I find period products to be unaffordable**		
Yes	4.62	3.10–6.89*
No (Reference)		
**I have struggled to purchase period products due to a lack of income/funds at some point in my life**		
Yes	2.09	1.37–3.19*
No (Reference)		
**I have personally experienced a period poverty situation**		
Yes	3.96	2.38–6.59*
No (Reference)		

* Statistically significant at 95% CI.

C-Statistic: 0.843.

### Struggling to access supplies over the past year

While 43% of respondents indicated that they had struggled to purchase period products at some point in their life, 29% said they had struggled in the past year. Finding period products to be unaffordable based on their income (aOR 6.27 95% CI 4.07–9.64) and struggling to purchase ever in their life (aOR 8.63 95% CI 5.39–13.83) were two statistically significant predictors for struggling to purchase in the past year. Having children under 18 (aOR 1.85 95% CI 1.20–2.85), making less than $30,000 a year (aOR 1.67 95% CI 1.03–2.71), and having a high school education or less (aOR 2.39 95% CI 1.34–4.26) were also statistically significantly associated with struggling to purchase products in the past year. This weighted regression model analyzed 922 complete cases (Table 4).

**Table 4:** Weighted logistic regression for predictors of struggling to purchase period products in the past year due to the COVID-19 pandemic (*n* = 922).

Variable	Adj. Odds Ratio	Confidence Interval [95%]
**Age**		
18–24	1.66	0.66–4.19
25–34	1.33	0.58–3.07
35–44	1.54	0.66–3.62
45–49 (Reference)		
Race/Ethnicity		
Black	1.49	0.77–2.89
Hispanic	0.99	0.59–1.65
Other	1.35	0.61–3.00
White (Reference)		
**Education Level**		
Less than high school or High School	2.39	1.34–4.26*
Some college or 2-year degree	1.26	0.73–2.17
4-year degree or higher (Reference)		
**Family Household Income**		
Under $30 k	1.67	1.03–2.71*
Over $30 k (Reference)		
**Partnership Status**		
Currently in partnership	1.12	0.68–1.79
Not currently in partnership (reference)		
**Children Under 18**		
Yes	1.85	1.20–2.85*
No (Reference)		
**Based on my income, I find period products to be unaffordable**		
Yes	6.27	4.07–9.64*
No (Reference)		
**I have struggled to purchase period products due to a lack of income/funds at some point in my life**		
Yes	8.63	5.39–13.83*
No (Reference)		
**I have personally experienced a period poverty situation**		
Yes	3.09	1.73–5.53*
No (Reference)		

* Statistically significant at 95% CI.

C-Statistic: 0.907.

### Missing work due to lack of supplies

The COVID-19 pandemic and the inability to afford and access period products were significantly associated with missing work. Overall, 18.4% of participants reported having to miss work, either virtual or in-person, during the prior 12 months due to a lack of access to period products (Table 1). Of the 18.4% who indicated yes, 58% missed in-person work, 29% missed virtual work, and 13% reported missing both virtual and in-person work. In a weighted regression model consisting of 943 complete cases, people aged 25–34 (aOR 6.34; 95% CI 1.813–22.14) and those with children under 18 (aOR 1.72; 95% CI 1.11–2.67) were significantly more likely to report missing work due to a lack of access to period products. Furthermore, those who stated that the pandemic made it more difficult to access period supplies were over four times more likely (aOR 4.32 95% CI 2.69–6.94) to report missing work. However, participants living in smaller households were less likely to miss work compared to those in households of 4 or more people (aOR 0.53 95% CI 0.35–0.81) (Table 5).

**Table 5:** Weighted logistic regression for predictors of missing work due to a lack of access to period products (*n* = 943).

Variable	Adj. Odds Ratio	Confidence Interval [95%]
**Age**		
18–24	3.14	0.84–11.73
25–34	6.34	1.813–22.14*
35–44	2.70	0.75–9.69
45–49 (Reference)		
**Race/Ethnicity**		
Black	1.48	0.79–2.78
Hispanic	1.07	0.65–1.76
Other	0.68	0.27–1.73
White (Reference)		
**Education Level**		
Less than high school or high school	0.79	0.45–1.41
Some college or 2-year degree	1.42	0.84–2.41
4-year degree or higher (Reference)		
**Household Size**		
1–3 people		
4 or more people (Reference)	0.53	0.35–0.81*
**Partnership Status**		
Currently in partnership	1.05	0.66–1.68
Not currently in partnership (reference)		
**Children Under 18**		
Yes	1.72	1.11–2.67*
No (Reference)		
**Based on my income, I find period products to be unaffordable**		
Yes	1.86	1.13–3.06*
No (Reference)		
**I have struggled to purchase period products due to a lack of income/funds at some point in my life**		
Yes	0.89	0.53–1.48
No (Reference)		
**I have personally experienced a period poverty situation**		
Yes	1.79	1.01–3.17*
No (Reference)		
**The Covid-19 Pandemic made it more difficult for me to access period products**		
Yes	4.32	2.69–6.94*
No (Reference)		
**I have struggled to purchase period products due to a lack of income/funds at some point in the past year**		
Yes	1.80	1.08–2.98*
No (Reference)		

* Statistically significant at 95% CI.

C-Statistic: 0.837.

## Discussion

This study aimed to investigate the effects of the COVID-19 pandemic on period poverty among adults in the U.S. through a nationally representative survey. Nearly one-third of the sample reported that the pandemic made it more difficult for them to access period products, and a similar percentage said they struggled to afford period products over the past year during the pandemic. These large percentages demonstrate the substantial impact the pandemic had on access to basic needs such as period products. Furthermore, having children under the age of 18 was significantly associated with all three major outcomes, which may reflect how the pandemic was particularly hard on these households that are especially vulnerable in times of economic downturn. Additionally, other predictor variables that reflect pre-pandemic struggles to access period products, such as ever experiencing period poverty and struggling to afford products ever in your life, were also significant results, which suggests the pandemic had an exacerbating effect on those already struggling to afford and access products.

Over half the sample indicated they have experienced a situation of period poverty. The framing of the question as “ever in your life” likely contributed to this high percentage, as individuals only needed to experience period poverty one time in order to respond “yes”. Occasionally having to use a substitute period product such as toilet paper or paper towels may have arisen from a lack of preparedness rather than a true inability to afford products. Therefore, finding period products to be unaffordable based on their income (38%) may provide a more accurate representation of current challenges those in the U.S. face.

Our results show a statistically significant association between struggling to access period products during the COVID-19 pandemic and Hispanic ethnicity, households with children, and lower incomes, which concurs with documentation of monthly poverty rates in the U.S. between February and September 2020 (23). As may be expected, those who struggled economically prior to the pandemic indicated additional challenges during COVID-19. Despite the economic benefits of the CARES Act, these households were hit particularly hard by the impacts of the pandemic, and this shows up in data around basic needs (14), including our data around accessing period products. With the closure of stores, childcare centers, and schools combined with the health risks of leaving home or using public transportation, many individuals may also have faced non-financial challenges to obtaining period products even though in this study only 4% of those who reported the COVID-19 pandemic made it more difficult for them to access period products indicated transportation barriers as a reason.

The disproportionate impact of the COVID-19 pandemic on lower-income menstruating individuals has been documented in countries like India (24). Recent data from the U.S. suggest that students' access to period products during the pandemic was negatively affected by school closures, especially for Latinx, rural, low-income, and college students (25). The percent of students who reported they have struggled to afford products (23%) (25) is similar to our results indicating that 29% struggled to purchase period products due to a lack of income/funds at some point in the past year. Our results among adults 18–49 show the impacts of the pandemic in the U.S. beyond just students. Like India, the U.S. experienced similar effects on lower-income populations, despite the country's higher overall GDP and broader economic resources at a national level. Those living in the U.S. with only a high school degree or lower level of education or a yearly income of less than $30,000 face similar challenges to affording products as those in lower-income countries.

In countries like Nigeria, the pandemic curtailed the ability of many non-governmental organizations to distribute menstrual hygiene products and education (26). Mass media stories from the U.K. and the U.S. during the early months of the pandemic reported a dramatic uptick in demand for period products from non-profit organizations that continued serving those in need (27). In France, a study conducted during the country's first and second pandemic lockdowns reported a significant relationship between period poverty and depression and anxiety (4). Here in the U.S., the shuttering of schools and transmission risks associated with public transportation early in the pandemic likely contributed to the increased rates of period poverty observed, especially during the first year of the pandemic. Given the substantial percentage of students who report relying on schools for access to period products (9, 11, 25), planning for future pandemic response must consider how to counteract the negative consequences of these necessary public health measures in order to minimize the ripple effects of a pandemic.

A smaller subset of our overall sample (18%) reported missing work due to lack of sufficient period products, with the majority of those who reported missing work aged 25–34. The association between missing work during the pandemic and struggling to access products because of the COVID-19 pandemic is consistent with findings from Sommer and colleagues who found that women who were out of work or unemployed reported more issues accessing period products (20). It is also consistent with data from prior to the COVID-19 pandemic in lower- and middle-income countries that highlight some of the challenges of managing menstruation in the workplace, including absenteeism (28, 29). Interestingly, several respondents in our study reported missing virtual or remote work due to a lack of period products. This effect on virtual/remote work should be explored further in future research as an increasing number of employers are codifying remote or hybrid work environments for their employees as standard. Given that higher paid jobs are more likely to be remote than low wage jobs, the relationship between income, period poverty, and missing work must be disentangled across the wage spectrum.

Reporting pre-existing challenges with access and affordability of period products prior to the pandemic was a significant predictor across all three outcome variables, contributing to higher proportions of respondents indicating that the pandemic made it harder for them to access products, that they struggled to afford products in the past year, and that they have missed work due to a lack of products. This finding shows how those already living in economically fragile situations were hit particularly hard by the COVID-19 pandemic which concurs with the findings from Sommer and colleagues (21) among a cohort of menstruating individuals already enrolled in a study about the pandemic.

### Limitations

This study has several limitations to note. First, as any secondary analyses are, we were limited to variables as provided in the dataset. For example, racial and ethnic identity were collected *via* a single variable instead of two as standard per the U.S. Census Bureau (30). Annual income and household size were categorical variables instead of ratio, limiting our ability to calculate federal poverty level thresholds. There were no variables about heads of household who might not be menstruating themselves but are buying products for others in their house. Similarly, there were no variables about education, stigma, facilities, or waste management so our analyses focus on the financial aspects of period poverty for those who are menstruating themselves. Next, respondents self-reported their experiences; there were no objective measures of their level of product insecurity. Furthermore, the cross-sectional nature of the study design limits us to testing associations. We cannot establish temporality or test causation with these data. Finally, given the self-reported, online nature of the survey administration, there may have been response bias in that only those with internet access are enrolled in the panel and those more interested in the topic may have been more likely to complete the survey. The sample weighting should have minimized this bias, however. Despite these limitations, this study represents one of the first nationally-representative looks at the impact of the COVID-19 pandemic on access to period products and period poverty in the U.S.

## Conclusion

Our findings illustrate a high-level of need for access to affordable period products during the COVID-19 pandemic, especially among Hispanics, those with children under 18, household incomes less than $30,000/year, and a high school education or less. These findings, highlighting the disproportionate impact of the COVID-19 pandemic on vulnerable households, point to the need for governments, organizations, and schools such as colleges and universities to include menstrual hygiene and period products in their pandemic preparedness and emergency response and relief planning. For example, they should consider how vital access to these products may need to shift to essential services and businesses when existing access points such as schools are forced to close, how to make basic needs such as period products available to those who must isolate or quarantine, and what communication will be made to ensure that the most vulnerable households are aware of how to access these basic needs during emergencies. From a policy perspective, our findings suggest that increasing affordability of products, such as through reducing or eliminating taxation on period products, and implementing supportive workplace policies, such as providing period products in restrooms just as toilet paper is provided or bathroom breaks as needed to manage menstruation, may help reduce menstruation-related work absenteeism. Finally, future research should focus on tracking trends in period poverty over time, utilizing qualitative methods to better understand how vulnerable households manage their basic needs during acute economic downturns and emergency situations such as lockdowns and isolation or quarantine, and developing validated measures of “period poverty” that capture a holistic perspective beyond just affordability and accessibility. These measures need to include access to facilities and waste disposal as well as education, awareness, and stigma around menstruation and be appropriate for the context of higher-income countries. We must learn from the COVID-19 pandemic and be better prepared to mitigate the effects of emergency situations on the most vulnerable individuals and households in our communities.

## Data Availability

The data analyzed in this study is subject to the following licenses/restrictions: the authors do not own the data. Alliance for Period Supplies owns the data. Requests to access these datasets should be directed to the corresponding author: Anne Sebert Kuhlmann, anne.sebertkuhlmann@slu.edu
